# Supplementary material for: Hypericin Ameliorates Depression-like Behaviors via Neurotrophin Signaling Pathway Mediating m6A Epitranscriptome Modification
Source: Molecules. 2023 May 3;28(9):3859. doi: 10.3390/molecules28093859 (PMC10179818; doi:10.3390/molecules28093859)
Supplement: Supplementary file 1 [file molecules-28-03859-s001.zip › molecules-2304407-supplementary.pdf]

Abbreviations

METTL3	Methyltransferase-like protein 3
METTL14	Methyltransferase-like protein 14
WTAP	Wt1 Associated Protein
FTO	Fat Mass and Obesity Associated Protein
ALKBH5	Alkb Homolog 5
Sirt1	Sirtuin 1
MDD	Major depressive disorder
WHO	World Health Organization
Adrb2	Adrenoceptor beta 2
MYC	Myc Proto-Oncogene
TCAs	Tricyclic antidepressants
SSRIs	Selective serotonin reuptake inhibitors
UCMS	Unpredictable chronic mild stress
SPT	Sucrose preference test
FST	Forced swimming test
TST	Tail suspension test
OFT	Open field test
NGF	Nerve growth factor
BDNF	Brain-derived neurotrophic factor
NT-3	neurotrophin-3
NT-4	neurotrophin-4
Akt3	AKT serine/threonine kinase 3
Ntrk2	Neurotrophic receptor tyrosine kinase 2
Braf	B-Raf proto-oncogene, serine/threonine kinase
Map2k1	Mitogen-activated protein kinase kinase 1
Kidins220	Kinase D interacting substrate 220
Shank1	SH3 and multiple ankyrin repeat domains 1
Syt7	Synaptotagmin 7
Igfbp5	Insulin like growth factor binding protein 5
CYP	P450 enzymes
Ndufs6	NADH:ubiquinone oxidoreductase subunit S6
Mef2d	Myocyte enhancer factor 2D
Nr2f2	Nuclear receptor subfamily 2 group F member 2
Rimbp2	RIMS binding protein 2
Gpx3	Glutathione peroxidase 3
Pcdhga11	Protocadherin gamma subfamily A, 11
Hspa9	Heat shock protein family A (Hsp70) member 9
Rab11fip5	RAB11 family interacting protein 5
Zbtb7a	Zinc finger and BTB domain containing 7A
Ncor2	Nuclear receptor corepressor 2
Muc6	Mucin 6, oligomeric mucus/gel-forming
Adcyap1r1	ADCYAP receptor type I

Supplementary Materials and Methods

1. Chronic unpredictable mild stress model and treatments

Four-week old C57BL/6 male mice were bred for 2 weeks and used in the subsequent experiments. All animal experiments were carried out in compliance with a protocol approved by the University Animal Care and Use Committee at SHUTCM (PZSHUTCM210608001) and in accordance with the guidelines of the National Institutes of Health Guide for the Care and Use of Laboratory Animals. All mice were housed under a 12 hours light/dark cycle at 25 ± 1 °C and were given standard diet and water ad libitum.

Unpredictable chronic mild stress (UCMS) model was established in mice according to a protocol described previously with minor revision. Briefly, mice in control group were housed with five per cage, but those of UCMS group were housed individually. The mice in the UCMS group randomly received 9 types of different stress for 21 days. These stresses mainly included tail pinch (2 min), food deprivation (24 h), bedding deprivation overnight, water deprivation (12 h), wet bedding (12 h), cage tilting of 45° (24 h), overnight illumination (12 h), noise (20 min) and body restraint (2 h). The mice of control group were housed under normal conditions. After administrated for three weeks, the mice of UCMS group were randomly assigned into three groups (n = 8 in each group): UCMS group, UCMS +Duloxetine (Duloxetine, 30 mg/kg, oral gavage) group, and UCMS +Hypericin (Hypericin, 2.4 mg/kg, oral gavage) group, also, the mice in the control group were treated with similar volume of saline.

2. Behavioral tests

2.1. Sucrose preference test (SPT)

The mice were acclimatized to two-bottle drinking for two days in advance. Two bottles contained 1% sucrose solution for the first day, and one bottle contained water and the other contained 1% sucrose solution for second day were drunk for mice. Mice were deprived for 24 h water and food before SPT. Next, the SPT was performed with two bottles (one is filled with water and other one with 1% sucrose solution) to the mice for 24 hours drinking, during which the position of each bottle was exchanged after 12 hours. The ratio of sucrose solution consumed was calculated by the formula (sucrose intake / (sucrose intake + water intake) × 100) as the sucrose preference.

2.2. Forced swimming test (FST)

The mice were individually placed in a glass bucket (height 30 cm, diameter 20

cm) which filled with water 25 ± 1 °C about 20 cm height. During the test, the mice can freely swim, and the immobility time was scored from video recordings of the last 4 min into the 6 min duration.

2.3. Tail suspension test (TST)

In TST, mice were suspended on a horizontal bar for the test duration of 6 min. Next, the immobility time was estimated the last 4 min no active movements.

2.4. Open field test (OFT)

For spontaneous locomotor activity in the OFT test, total distance of autonomous movement in the box and the time spent in the center area was recorded with the EthoVision video tracking system (Noldus Information Technology™, Leesburg, Virginia, USA). Each mouse was individually placed in the center of an open field (50 × 50 × 50 cm) and observed for 5 min in the open field area.

3. qRT-PCR

3.1 RNA extraction

The frozen hippocampal tissues of mice were taken separately from -80°C refrigerator, including control group, UCMS group and hypericin group. It was placed into 1.5 ml centrifuge tubes, added 500 μl Trizol per tube, and crushed it using ultrasound at 30% power, sonicated it for 15 s, and stopped it 4 times for 10 s. The tissue was then removed from the tube. This procedure was performed on ice. Then, centrifuge at 4°C, 12,000 rpm for 15 min. Aspirate 500 μl of supernatant into a new centrifuge tube, add 100 μl of chloroform, shake vigorously for 3 min and leave for 3 min. Then, centrifuge for 15 minutes at 4°C and 12000 rpm, aspirate 300 μl of the supernatant into a new 1.5 ml centrifuge tube, add 300 μl isopropanol, mix upside down and let stand for 10 minutes. Then, centrifuge for 15 minutes at 4°C and 12000 rpm when white RNA flakes are visible at the bottom of the tube. Wash with 500 μl of freshly prepared 75% ethanol and shake gently until the white precipitate floats up. Then, centrifuge for 10 min at 4°C and 12,000 rpm and pour off the ethanol. Repeat the washing procedure once with 75% ethanol. After pouring off the ethanol, allow the RNA to air dry at room temperature. The dried RNA will appear colorless and transparent. The concentration of RNA was measured using a NanoDrop 2000 ultra-micro spectrophotometer.

3.2 Reverse transcription cDNA

Using the kit for reverse transcription (HiScript II 1st Strand cDNA Synthesis Kit,

Vazyme, R211-01/02), 1 μg of total RNA was quantified for reverse transcription according to the RNA concentration as per the instructions for use. The 20 μl mixture (4 × gDNA wiper mix 4μl, 1μg total RNA, RNase-free ddH_2O) was first prepared, bubbled and mixed, and then, reacted in the PCR apparatus at 42°C for 5 min. Then, 4 μl of 5 × HiScript III qRT SuperMix was added and reacted in the PCR instrument at 37°C for 15 min followed by 85°C for 5 s.

3.3 qPCR

The qPCR assay was performed according to the kit instructions for use (AceQ Universal SYBR qPCR Master Mix, Vazyme, Q511-02/03). The reaction system was configured as follows: 5 μl 2 × AceQ Universal SYBR qPCR Master Mix, 0.2 μl Primer1(10 μM), 0.2 μl Primer2(10 uM), 3.6 μl ddH2O and 1ul cDNA. Amplification was performed in the qPCR reaction instrument. Reaction conditions were 95 ℃, 5 min for predenaturation, followed by 40 cycles of 95 ℃, 10 s; 60 ℃, 30 s. Among them, 95 ℃, 15 s; 60 ℃, 60 s; 95 ℃, 15 s were the reaction conditions for the lysis curve. β-ACTIN gene was used to the internal control and the $2^{\wedge(-\Delta\Delta Ct)}$ methods was calculated the relative level of each mRNA.

4. Western blot

4.1 Protein extraction

The frozen hippocampal tissues of the mice were taken separately from the -80°C refrigerator, including the control group, UCMS group and hypericin group. Place them into a 1.5 ml centrifuge tube and added 300 ul protein lysate (RIPA + protein inhibitor + phosphatase inhibitor) to each tube. Comminution was then performed by sonication at 30% power, 15 s sonication with 10 s pause, 4 times, and this procedure was performed on ice. Centrifugation was then performed at 4°C and 12,000 rpm for 15 min. The precipitate was discarded and the supernatant was transferred to a new 1.5 ml centrifuge tube.

4.2 Protein concentration determination

The instructions for use of the BCA quantification kit were followed and the average of three replicate wells was used as the final reading for each sample. Preparation of standard curve: In a 96-well plate, the standard curve was prepared in a concentration gradient of 0, 0.1, 0.2, 0.4, 0.8, 1.2, 1.6, 2.0 μg/μl and quantified on 20 μl with ddH_2O. Sample preparation: 2 μl of the sample to be tested is added to each well and quantified to 20 μl with ddH_2O. According to the instructions of the BCA kit, the working solution (ready-to-use) was prepared by mixing liquid A and liquid B in a

50:1 ratio. 200 μl of the working solution was added to each well, covered and placed on a shaker at 37℃, protected from light and incubated at 400 rpm for 30 min. Combined with the standard curve, the protein concentration of the sample to be measured was calculated. Protein samples (1 μl/μg) were prepared in a 20 μl system using 5×SDS loading buffer and lysate as diluent according to the protein concentration. incubated at 95°C for 15 min and stored at -20°C after cooling.

4.3 Sodium dodecyl sulfate polyacrylamide gel electrophoresis (SDS-PAGE)

Next, a total of 20 ug of protein from each sample was separated by electrophoresis in 10% and 12% SDS-PAGE gels. The proteins were then transferred to a polyvinylidene fluoride (PVDF) membrane and blocked with a buffer containing 5% nonfat milk for 2 hours and incubated overnight at 4°C with primary antibodies. The primary antibodies were listed as follows: anti-METTL3 (1:2000, Proteintech, 15073-1- AP), anti-FTO (1:2000, Proteintech, 27226-1- AP), anti-WTAP (1:5000, Proteintech, 60188-1-Ig), β-ACTIN (1:50000, Proteintech, 81115-1-RR). Subsequently, the proteins were hybridized with the secondary antibodies for 1 h and protein binding was detected by chemiluminescence methods.

5. RNA-seq

5.1 Library construction and quality control

Total mRNA was extracted with oligo (dT) from 2 μg total RNA. Subsequently, the mRNA was fragmented to a size of 200-300 bp using fragmentation buffer. The reaction conditions for fragmentation were 94°C for 5 min and 4°C for pause. The fragmented mRNA served as a template and random oligonucleotides were used as primers to synthesize the first strand of cDNA in the 1st Strand Enzyme Mix Reverse Transcriptase System. The RNA chain was then degraded with RNase H and the second strand of cDNA was synthesized with dNTPs under the 2nd Strand Enzyme Super Mix 2 system. The purified double-stranded cDNAs were repaired at the end, A-tailed, and ligated to sequencing connectors. The cDNAs were screened with DNA clean beads, PCR-amplified (12 cycles), and the PCR products were purified again with DNA clean beads to obtain libraries. The quality of the libraries was reviewed and quantified using the BioAnalyzer 2100 system (Agilent Technologies, Santa Clara, California, USA) and sequenced using the Illumina Novaseq platform. After the library was prepared, it was first quantified using a Qubit 2.0 fluorometer and diluted to 1.5 ng/μl. The insert size of the library was then measured using an Agilent 2100 Bioanalyzer, and the effective concentration of the library was accurately determined by qRT-PCR after the insert size

met expectations (effective library concentration above 2 nM) to ensure library quality.

5.2 Data Analysis

First, the raw data were filtered. It mainly includes removing reads with adapters, removing reads containing N (N means base information cannot be determined), and removing low-quality reads. Meanwhile, Q20, Q30 and GC content were calculated for clean data. All subsequent analyses were performed based on the clean data with high quality. The mouse gene annotation files and the reference genome indexed by Hisat2 (v2.0.5) were downloaded and then compared with the genome. After comparison, reads for each gene were statistically calculated using featureCounts (1.5.0) to obtain the counts file for that gene. Differential analysis of counts files was performed using DEseq2, and KEGG pathway enrichment analysis and GO analysis of differential genes were performed using KOBAS.

6. m6A immunoprecipitation

The m6A RNA Enrichment Kit (Epigentek, Farmingdale, Illinois, USA) was applied to perform the MeRIP assays in line with the manufacturer's protocols.

6.1 Immunocapture and Cleavage

Prepare 200 ul of Immunocapture Solution by adding the reagents to 0.2 ml PCR tubes (174-189 µl of Immuno Capture Buffer, 2 µl of m6A antibody, 4 µl of affinity protein G beads and 10 µg of total RNA) and mix well. Spin the tube on a rotator or shaker at room temperature for 90 minutes. The RNA was then cleaved with a cleavage solution (Nuclear Digestion Enhancer and Cleavage Enzyme Mix). The immunoprecipitation complex was then washed three times with wash buffer.

6.2 Enriched RNA Release/Recovery

Prepare the protein digestion solution by mixing Proteinase K with PDB (Protein Digestion Buffer) at a ratio of 1:10 and adding 20 µl of the protein digestion solution to the immunoprecipitation complex. Mix and incubate at 55°C for 15 min in a thermal cycler to detach the enriched RNA from the magnetic beads. Place the tubes on the magnetic device until the solution is clear (approximately 2 min). Carefully transfer the solution from each sample to an unused PCR tube. Add 20 µl of the RPS (RNA Purification Solution) to each sample, followed by 160 µl of 100% ethanol. Immediately after, 2 µl of RNA binding beads were added and mixed to capture the RNA fragments. The magnetic beads were then washed twice with 150 µl of freshly prepared 90% ethanol. Finally, the enriched RNA was eluted with 10 µl of elution buffer and stored at -20°C for subsequent sequencing and qPCR experiments.

7. MeRIP-seq

7.1 Library construction and quality control

The fragmented RNA obtained from the above m6A immunoprecipitation experiment served as template and random oligonucleotides were used as primers to synthesize cDNA in the SMARTScribe Reverse Transcriptase Reverse Transcriptase System. The PCR reaction system (5 cycles) with SeqAmp DNA ploymerase was followed by the addition of lllumina adapters and barcodes to both ends of the cDNA, followed by purification of the PCR products with AMPure XP beads. Subsequently, ribosomal cDNA was removed using ZapR v2 and R-Probes v2. Finally, the library was amplified with 12 cycles and AMPure XP beads were used to purify the final RNA-seq library. The quality of the libraries was checked and quantified using the BioAnalyzer 2100 system (Agilent Technologies, Santa Clara, California, USA) and sequenced using the Illumina Novaseq platform. After the library was prepared, it was first quantified using a Qubit 2.0 fluorometer and diluted to 1.5 ng/μl. The insert size of the library was then measured using an Agilent 2100 Bioanalyzer, and the effective concentration of the library was accurately determined by qRT-PCR after the insert size met expectations (effective library concentration above 2 nM) to ensure the quality of the library.

7.2 Data analysis

Raw data (raw reads) of fastq format were firstly processed using fastp (version 0.19.11). In this step, clean data (clean reads) were obtained by removing reads containing adapter, reads containing ploy-N and low-quality reads from raw data. At the same time, Q20, Q30 and GC content the clean data were calculated. All the downstream analyses were based on the clean data with high quality. The mouse reference genome and gene annotation files were then downloaded, a reference genome index was generated using STAR (Spliced Transcripts Alignment to a Reference), and genomic comparisons were performed. After mapping the reads to the reference genome, the R package exomePeak (version 2.16.0) was used to identify m6A peaks in each anti-m6A immunoprecipitation group, using the corresponding input samples as controls. The m6A-enriched motifs of each group were identified using HOMER (version 4.9.1). Differential peak analysis was performed using MeTPeak (MeTPeak, San Antonio, Texas, USA), and KEGG pathway enrichment analysis and GO analysis were performed using the genes corresponding to the differential peaks. Next, ChIPseeker was used to examine the peaks in the genomic functional regions, including the promoter, 5' UTR, 3' UTR, exon, and intron regions, and annotate the functions of

the peaks with TxDb.Mmusculus.UCSC.mm10.knownGene.

8. MeRIP-qPCR

m6A immunoprecipitation was consistent with experiment 4 above. Random primers are used for reverse transcription, and the rest of the steps are the same as in experiment 1.3 above. Additionally, the primers were designed according to the m6A peak found in differential peak analysis. Amplification was performed in the qPCR reaction instrument. Reaction conditions were 95 ℃, 5 min for pre-denaturation, followed by 40 cycles of 95 ℃, 10 s; 60 ℃, 30 s. Among them, 95 ℃, 15 s; 60 ℃, 60 s; 95 ℃, 15 s were the reaction conditions for the lysis curve. The mRNA of the corresponding sample without m6A immunoprecipitation was used to the internal control. Additionally, the $2^{\wedge(-\Delta\Delta Ct)}$ methods was calculated the relative level of each mRNA.

Supplementary Table S1. Primers for qPCR and MeRIP-qPCR.

Genes	Forward primer	Reverse primer
NTRK2	GTTTGGCATGAAAGGCCCAG	GTCTGGCTTGAGCTGACTGT
MAP2K1	GCTTCTACGGGGCCTTCTAC	CTCCCGAAGATAGGTCAGGC
AKT3	GTGGGGCTTAGGTGTTGTCA	CCCTCCACCAAGGCGTTTAT
BRAF	CCCACAGATGCATCACGGA	CACATCTTGCGGGTACCACT
KIDINS220	CATGTGGGCGTGCTACAAAG	GGGTAGACACTGTACAAACCAGT
CAMK4	CCCTGGTGGGATGAAGTGTC	ATGTGAACAAAGTTGGCCGC
ARHGDIG	ATCACACCTATCGTCGGGGA	AGTGAAGAGGGACCTGACCA
SORT1	GCGGAAGAGTGTTCAGGTCA	AGGAGCCATTCACATGGGTG
β-ACTIN	GGCTGTATTCCCCTCCATCG	GCTTCTACGTGTGTGCTTTCG
NTRK2 Gene-specific m6A qPCR	TGCAGCTAAGTGACAGCTCC	TCGGGGGTCAAGATTTCTGC
AKT3 Gene-specific m6A qPCR	ACATCCATCCCTTGGCACTG	TGGTAAGGCTCTGGGACTGT
BRAF Gene-specific m6A qPCR	GTAACTCACAGCCCAGCCAA	AGCACATGAGGGGTGTGTTT
KIDINS220 Gene-specific m6A qPCR	GGTCTGCGCTACCAGAAACT	TCTCTCTGCTTTGCCTTCGG
MAP2K1 Gene-specific m6A qPCR	CCCGGTCTTTCCTGAAACGTA	GGCACATGTGTACCCACAGA

Supplementary Table S2. The top DEGs identified from UCMS vs. control mice.

Gene	Description	p-value	Log2FC
Shank1	SH3 and multiple ankyrin repeat domains 1	2.74E-07	0.359
Syt7	Synaptotagmin 7	3.38E-07	0.354
Igfbp5	Insulin like growth factor binding protein 5	6.64E-07	0.470
Pcdh1	Protocadherin 1	1.02E-06	0.496
Prepl	Prolyl endopeptidase like	2.98E-06	0.597
Gprc5b	G protein-coupled receptor class C group 5 member B	6.00E-06	0.319
Dbp	D-box binding PAR bZIP transcription factor	6.57E-06	0.489
Nr1d1	Nuclear receptor subfamily 1 group D member 1	9.65E-06	0.341
Rab11fip4	RAB11 family interacting protein 4	2.32E-05	0.608
Ndufs6	NADH:ubiquinone oxidoreductase core subunit S6	2.56E-05	-0.430

Supplementary Table S3. The top DEGs identified from overlapping of UCMS vs. control mice and UCMS vs. hypericin treatment.

Genes	Description	UCMS vs. Control		UCMS vs. Hypericin	
		p-value	Log2FC	p-value	Log2FC
Ndufs6	NADH:ubiquinone oxidoreductase core subunit S6	0.00003	-0.43	0.00553	-0.39
Mmp14	matrix metallopeptidase 14	0.00008	-0.55	0.01931	-0.34
Mef2d	myocyte enhancer factor 2D	0.00017	0.30	0.03817	0.25
Ctxn3	cortexin 3	0.00019	1.37	0.04659	0.71
Nr2f2	nuclear receptor subfamily 2 group F member 2	0.00025	-0.45	0.04856	-0.32
Rimbp2	RIMS binding protein 2	0.00033	0.32	0.02707	0.33
Agt	angiotensinogen	0.00036	-0.42	0.00000	-0.70
Gpx3	glutathione peroxidase 3	0.00056	-0.76	0.01943	-0.43
Pcdhga11	protocadherin gamma subfamily A, 11	0.00149	0.88	0.04213	0.57
Necab2	N-terminal EF-hand calcium binding protein 2	0.00188	-0.26	0.00015	-0.41

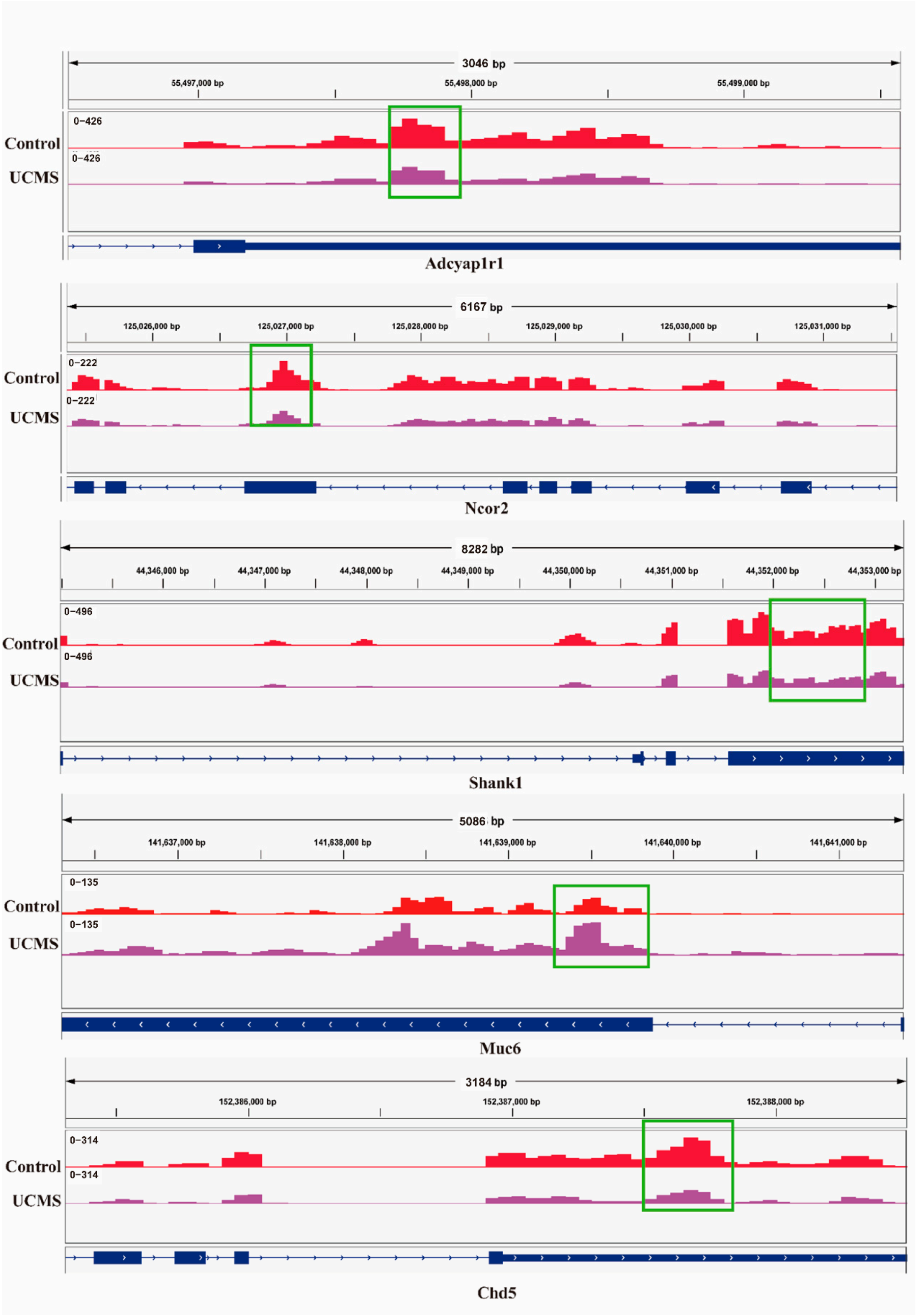

Supplementary Figure S1. Abundant m6A in Ncor2, Shank1, Muc6, Adcyap1r1 and Chd5 mRNA transcripts in control and UCMS treatment.

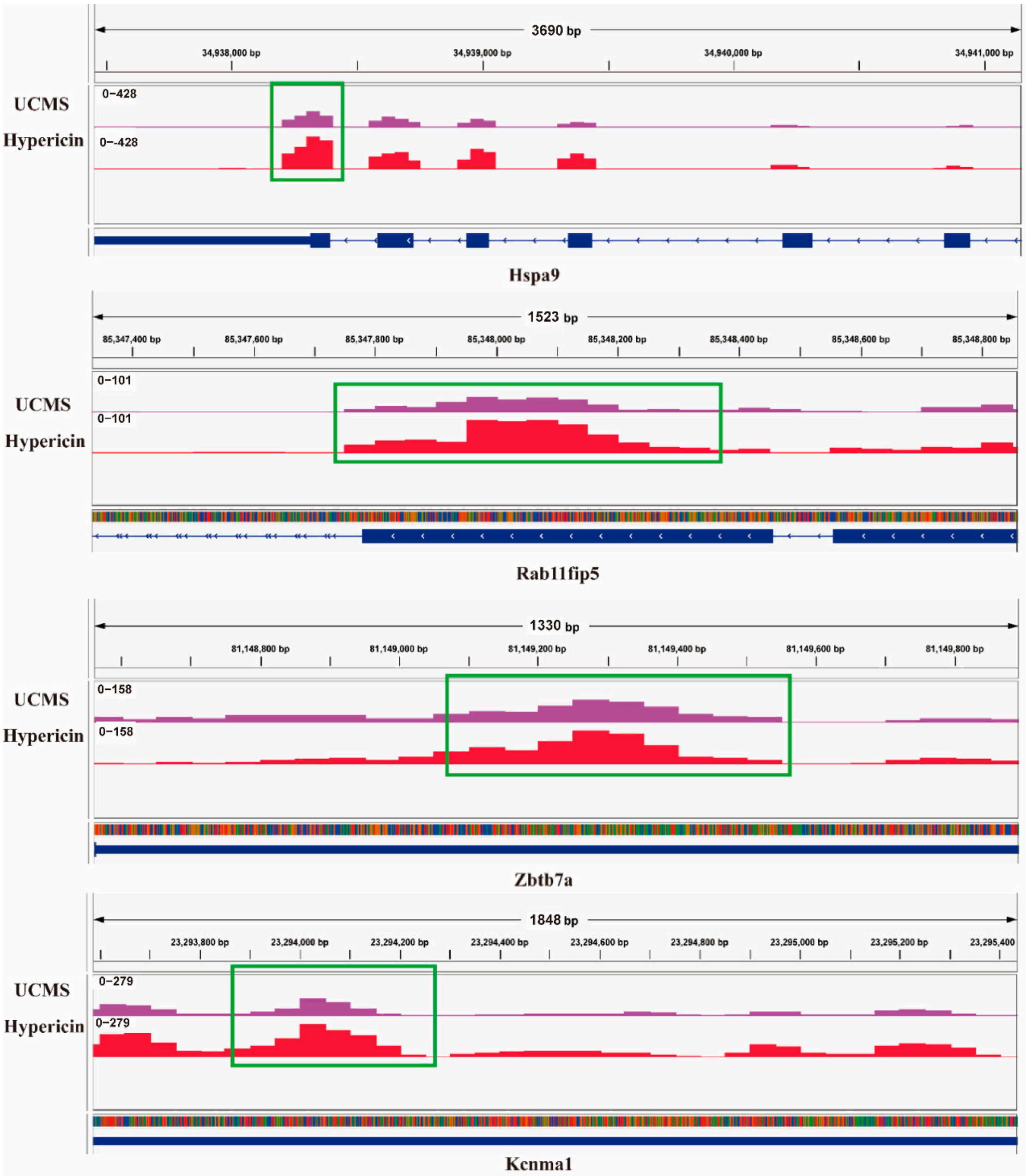

Supplementary Figure S2. Abundant m6A in Hspa9, Rab11fip5, Zbtb7a and Kcnma1 mRNA transcripts in UCMS and hypericin treatment.
